# Discovery and characterization of 91 novel transcripts expressed in cattle placenta

**DOI:** 10.1186/1471-2164-8-113

**Published:** 2007-05-09

**Authors:** Charu G Kumar, Joshua H Larson, Mark R Band, Harris A Lewin

**Affiliations:** 1 Laboratory of Mammalian Genome Biology, Department of Animal Sciences, University of Illinois at Urbana-Champaign, 210 Edward R. Madigan Laboratory, 1201 W. Gregory Dr., Urbana, IL 61801, USA; 2 The W.M. Keck Center for Comparative and Functional Genomics, University of Illinois at Urbana-Champaign, 356 Edward R. Madigan Laboratory, 1201 W. Gregory Dr., Urbana, IL 61801, USA; 3 Institute for Genomic Biology, University of Illinois at Urbana-Champaign, 1206 West Gregory Drive, Room 1608, Urbana, IL 61801, USA

## Abstract

**Background:**

Among the eutherian mammals, placental architecture varies to a greater extent than any other tissue. The diversity of placental types, even within a single mammalian order suggests that genes expressed in placenta are under strong Darwinian selection. Thus, the ruminant placenta may be a rich source of genes to explore adaptive evolutionary responses in mammals. The aim of our study was to identify novel transcripts expressed in ruminant placenta, and to characterize them with respect to their expression patterns, organization of coding sequences in the genome, and potential functions.

**Results:**

A combination of bioinformatics, comparative genomics and transcript profiling was used to identify and characterize 91 novel transcripts (NTs) represented in a cattle placenta cDNA library. These NTs have no significant similarity to any non-ferungulate DNA or RNA sequence. Proteins longer than 100 aa were predicted for 29 NTs, and 21 are candidate non-coding RNAs. Eighty-six NTs were found to be expressed in one or more of 18 different tissues, with 39 (42%) showing tissue-preference, including six that were expressed exclusively in placentome. The authenticity of the NTs was confirmed by their alignment to cattle genome sequence, 42 of which showed evidence of mRNA splicing. Analysis of the genomic context where NT genes reside revealed 61 to be in intergenic regions, whereas 30 are within introns of known genes. The genes encoding the NTs were found to be significantly associated with subtelomeric regions.

**Conclusion:**

The 91 lineage-specific transcripts are a useful resource for studying adaptive evolutionary responses of the ruminant placenta. The presence of so many genes encoding NTs in cattle but not primates or rodents suggests that gene loss and gain are important mechanisms of genome evolution in mammals. Furthermore, the clustering of NT genes within subtelomeric regions suggests that such regions are highly dynamic and may foster the birth of novel genes. The sequencing of additional vertebrate genomes with defined phylogenetic relationships will permit the search for lineage-specific genes to take on a more evolutionary context that is required to understand their origins and functions.

## Background

The primary function of the placenta is to regulate the transport of gases, nutrients and waste products between mother and fetus [1]. The placenta also serves as an endocrine organ, producing estrogens, progesterone and placental lactogens that are important for the maintenance of pregnancy [1]. While these functions have been conserved in all eutherian mammals, the relatively large variation in placental architecture [2] makes the placenta an attractive model system for studying adaptive evolutionary changes [3]. Placentae are classified on the basis of their gross shape and the distribution of contact points between the fetal tissues and the maternal endometrium [2]. The *synepitheliochorial cotyledonary *placenta of ruminants has three distinct tissue layers and is regarded as the most complex as compared to other placental types [2]. By contrast, in the *discoid hemochorial *placenta of primates and rodents, the fetal chorionic epithelium is directly bathed in maternal blood because the three maternal tissues layers are degraded. The molecular basis for these anatomical changes and their adaptive significance remain largely unknown.

Recent studies demonstrating that phenotypic changes with adaptive significance can be caused by the action of individual genes provide an important rationale for the identification of genes that may be highly divergent or unique to a specific lineage or clade [eg, [4]]. Furthermore, there is a relative abundance of novel or lineage-specific transcripts (NTs) [5] and lineage-specific regulatory non-coding RNAs (ncRNAs) [6-8] in the transcriptomes of different eukaryotes. However, little is known about the role of divergent genes and lineage-specific transcripts in adaptive evolution. In ruminants, there is unequivocal evidence for lineage-specific and highly divergent genes expressed in the placenta and/or trophoblast e.g., genes encoding *interferon-tau *[9], the *placental lactogens *[10], the *pregnancy associated glycoproteins *[11] and the *prolactin related proteins *[12]. All of these highly divergent proteins appear to play adaptive roles in the reproductive biology of ruminants.

Given the unique adaptations of the reproductive system of ruminants, our goal is to use comparative genomics to identify genes and ncRNAs that are responsible for these evolutionary changes. Toward that end, we recently developed a bioinformatics strategy to mine collections of expressed sequence tags (ESTs) for divergent homologs and novel transcripts [13]. This strategy led to the discovery of the *ULBP *gene cluster and eight divergent homologs in cattle [12,14]. Herein, we describe the application and extension of our approach for identifying and characterizing NTs expressed in mammalian tissues. Using this approach, 91 NTs were identified in a collection of cattle placenta ESTs and then verified by *in silico *extension with DNA sequences in the public domain databases, gene expression profiling, and alignment to whole genome sequence.

## Results

### Identification and characterization of novel transcripts in cattle placenta

A collection of 12,614 5' ESTs from a cattle term placenta cDNA library was reduced to a working set of 373 putative NTs and divergent homologs using pairwise BLASTN [15] searches against non-cetartiodactyl EST and genome databases (October 2005 freezes), followed by *in silico *extension and full-clone sequencing of cDNA inserts (Table 1). Analysis of the sequence-extended EST-containing clones using TBLASTX searches against human and mouse UniGene [16], and against ESTs from non-cetartiodactyl species, permitted the distinction of divergent (N = 75) from unknown (N = 298) transcripts (Table 1). Repetition of *in silico *extension of EST sequences followed by removal of homologs using subsequently updated databases (April 2006 freezes) removed an additional 134 ESTs, thus leaving 164 putative NTs. Using the 6.2× Btau_2.0 cattle genome assembly [17] as a reference, 73 transcripts were found likely to represent priming from poly A tracts of genomic DNA. These artifacts were subsequently removed, bringing the final working set to 91 NTs that have an average length of 993 bp (Table 1; Additional file 1). The definition of a transcript as novel thus indicates that the nucleotide sequence or hypothetical proteins encoded by it does not have similarity to any non-cetartiodactyl DNA or protein sequence at the time the databases were searched. The operational term is not meant to imply an evolutionary mechanism, such as gene loss or rapid divergence.

Alignment to the cattle genome sequence assembly allowed polyA signals to be identified within 50 bp downstream from the end of the aligned NT. Polyadenylation signals were found in 86/91 (95%) of the NTs (Additional file 2). The consensus signal, AATAAA, was present in 54 (59%) of the NTs; 15 (16%) had the less conserved signal ATTAAA, and 17 (19%) had rare polyadenylation signals experimentally identified in human mRNA [18]. A polyadenylation signal was not detected in 5 (5%) of the NTs, likely representing incomplete transcripts.

**Figure 1:**
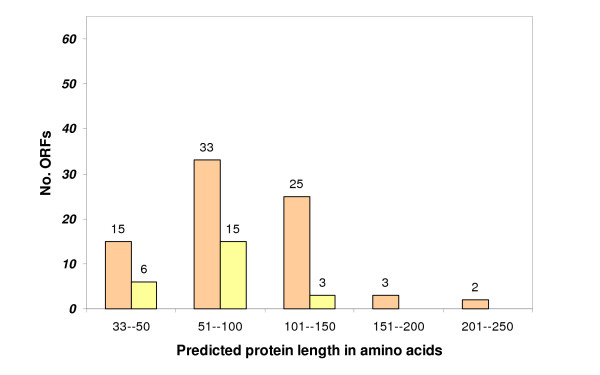
**Length distribution of ORFs**. Length distribution of 78 ORFs (orange bars) predicted in 64 NTs with >33 codons. The expected frequency of false positives (yellow bars) was calculated according to Frishman et al. [52].

Analysis of the 91 NTs revealed 64 NTs with one or more open reading frames (ORFs) >33 codons (Figure 1). Among these 64 NTs there are 78 predicted ORFs >33 codons, all of which were considered as candidates for encoding novel proteins (Figure 1; Additional file 3). The cDNA clone with the longest ORF, BTC1_14RD, contains 235 codons. TBLASTN of the translated ORFs against all predicted cattle transcripts resulted in 24 unique hits, of which seven are to hypothetical proteins, 15 are to predicted proteins (having some supporting molecular evidence), and two have similarity to known cattle proteins (Additional file 2). While all ORFs >33 codons terminate in a stop codon, 19 (21%) do not possess an ATG start codon, suggesting that at least some of the NTs represent 3' regions of genes or have ORFs with alternate start codons. The ORFs possessing an ATG start codon were analyzed for the presence of the Kozak consensus sequence RMC-ATG-G, a signal for eukaryotic translation initiation [19], where R is a purine and M is [AC]. One ORF matched this consensus pattern and 24 ORFs matched the less restricted Kozak pattern R-N-N-ATG-R, where N is any nucleotide, and R is a purine.

Protein motifs are predicted in the translated ORFs of four NTs (Additional file 2, Table 2). In addition, a transmembrane helix (TMH) was identified in one ORF (BTC1_403NG) predicted to contain a single-span TMH. Signal peptides are predicted in four ORFs with no concomitant TMH prediction (Additional file 2) thus indicating that these transcripts encode soluble/secreted proteins. Functional elements located in untranslated regions (UTRs) are predicted in 25 NTs (Additional file 2, Table 2). The NT BTC1_43PW contains a predicted selenocysteine insertion sequence (SECIS) element. These elements are required for translation of the UGA codon as selenocysteine in mRNAs of selenoproteins, several of which are species-specific and participate in peroxide degradation and antioxidant reactions [20]. The UTR sequence region corresponding to the SECIS prediction was manually verified for the appropriate secondary structure (Additional file 4). Two sequences, BTC1_40PW and BTC1_14RD, are predicted to contain conserved ncRNA secondary structural elements within their 3' UTRs thus implicating them in regulatory functions.

The GC-content average of the NTs, mRNAs and genomic DNA sequence is 0.50 (range 0.33 to 0.7), 0.51, and 0.45, respectively (Additional file 2). Ten percent of NTs have low GC content average (<0.4) as compared to 12% of full length mRNAs and 31% of genomic scaffold sequence that contains the NTs. Candidate exonic CpG islands were identified in 5' ends of four NTs (Table 3, Additional file 2). Criteria for CpG islands were length >200 bp, GC content ≥ 53% and observed/expected CpG ratio ≥ 0.63 [21]. Anchoring the NTs to the 6.2× draft cattle genome sequence allowed the identification of 26 additional NTs with CpG islands upstream of their start sites. Other NTs with regulatory sequence features included 12 with inverted repeats of at least 11 bp. The longest inverted repeats are 18 bp and separated by approximately 300 bp. In addition, 7 NTs contain G-quartets, which are tetrads of guanine/purine tetramers that are implicated in transcription pausing, mRNA stability, recombination hotspots, stability of chromosomes and interactions of telomeres [reviewed in [22]].

Twenty-one NTs qualified as candidate ncRNAs (Table 3, Additional file 2). These NTs have no ORF >33 codons, and no predicted exon within 5 kb of flanking cattle genome sequence. Among these, 20 contain a known polyadenylation signal, of which seven are spliced, and 14 align to the genome sequence along the transcript's entire length with >95% identity (Additional file 2).

### Genomic context of the NTs

The genomic organization of the NT genes was determined by BLASTN against the 6.2× cattle genome draft sequence. All 91 NTs have matches in the cattle genome (Additional file 2), 49 of which have ≥98% identity over >95% of their length and 48 have polyadenylation signals (intronless transcripts). The remaining 42 NTs have well-defined intron-exon boundaries indicative of mRNA splicing (an average of 3 exons), of which 40 possess a polyadenylation signal (Table 4; Additional file 2). CpG islands were located upstream of 16 of these 40 transcripts. Of the 91 NTs, 68 were mapped *in silico *to chromosome locations in the cattle genome on the basis of existing radiation hybrid (RH) and comparative mapping information [23] (Additional file 2). The remaining 23 were located on unmapped scaffolds. Among the 68 mapped NTs, 10 were located on BTA19 (P > 0.05). This analysis also revealed that 61 (67%) of the NTs are located within "intergenic" regions and 30 (33%) of the NTs are within introns of known genes (Table 3, Additional file 2). Three of the NTs on BTA19 (BTC1_390NG, BTC1_28PW, BTC1_8NG) were located within introns of the developmentally regulated genes *HOXB3*, *JAF1 *and *ATP1a2a*. In addition, 24 NTs (26%) are located either in subtelomeric regions or at the boundaries of homologous synteny blocks as defined by Everts van der Wind et al. [23] (Table 3; Additional file 2). The distribution of NTs was positively associated (P < 0.05) with the subtelomeric regions (< 2 Mbp from telomere) as compared to the distribution of randomly chosen RefSeq genes.

Among the 91 NTs, 71 have no NCBI [24] annotation. The 20 annotated NTs that aligned to the cattle genome were analyzed in greater detail. Each of them was found to have a gene model and a cattle RefSeq identification number (Additional file 2). Most appear to be either alternatively spliced variants, antisense to the predicted genes, or long and divergent 3' ends of predicted genes. The 20 annotated NTs were examined further by comparing full-length mRNA sequence from the cattle RefSeq prediction to the human genome. Similarity between the full-length cattle RefSeq predictions and human genes was found only for 12 of the NT-containing RefSeq genes (BLASTN E value < 10^-10^). Among these 12 cattle RefSeq genes with putative human orthologs, the NTs contained within them represent novel splice products, whereas three are novel antisense products. *In silico *comparative mapping confirmed homologous positions within the cattle and human genomes, thus providing additional supporting evidence that these 12 NTs are part of highly divergent genes and/or genes created *de novo *(Additional file 2).

**Figure 2:**
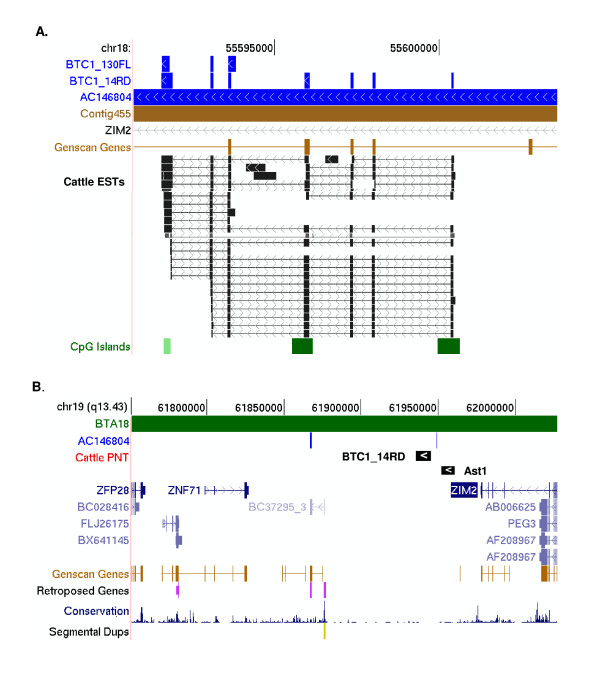
**Genomic context of BTC1_14RD and BTC1_130FL**. Top panel (A). A modified image from the UCSC cow genome browser (March 2005, Btau_2.0) showing BTC1_14RD and its alternately spliced product BTC1_130FL aligned to the cattle genome (Contig455) [GenBank:AAFC02000448]. Cattle BAC AC146804 [25] aligned to the same region using BLAT (regions of similarity shown with vertical bars). A track for human proteins is shown to demonstrate that there are no known human homologs in this region. A scaled track for cattle ESTs (partial representation of "squish mode" due to the large number of ESTs) shows high support for alternatively spliced cattle transcripts encoded in this region. Bottom panel (B). A modified image from the UCSC human genome browser (May 2004, Hg17) showing the *in silico *anchoring of BTC1_14RD, represented as a block arrow, to a subtelomeric region of HSA19q on the basis of flanking sequence similarity in cattle BAC AC146804. This region is syntenic to a segment of BTA18 (shown as a separate track at the top). BTC1_130FL anchors in the same region (not shown to maintain clarity of the figure). The assumptive map location of the gene encoding the artiodactyl-specific transcript *Ast1 *[GenBank:AY427788] is also shown. No significant flanking match was identified in Contig455 (due to its shorter length). Unmodified UCSC Genome Browser tracks for known human proteins, Genscan genes, retroposed genes, conserved sequences, and segmental duplication are shown.

**Figure 3:**
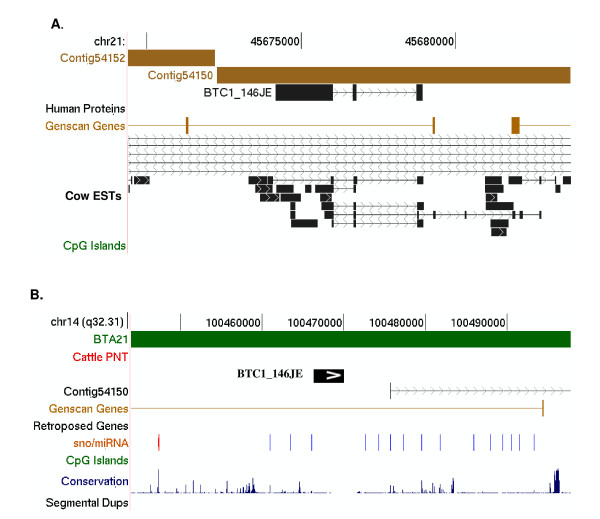
**Genomic context of BTC1_146JE**. Top panel (A). A modified image from the UCSC cow genome browser (March 2005, Btau_2.0) showing BTC1_146JE aligned to the cattle genome (Contig54150) [GenBank:AAFC02053608]. Cow ESTs are shown in a scaled "squish" mode to conserve space due to the large number of ESTs aligning in the region. Bottom panel (B). Anchoring of BTC1_146JE, represented as a *block arrow*, to HSA14q32.31 (May 2004, Hg17). BTC1_146JE was anchored on the basis of its complete alignment to contig54150 and similarity of flanking sequence in the contig to the human genome shown as a vertical line along the track. This region is syntenic to a segment of BTA21 (shown as a separate track), consistent with RH mapping data [23]. UCSC Genome Browser tracks for known human proteins, Genscan genes, sno/miRNA, conserved sequences, and segmental duplication are unmodified.

**Figure 4:**
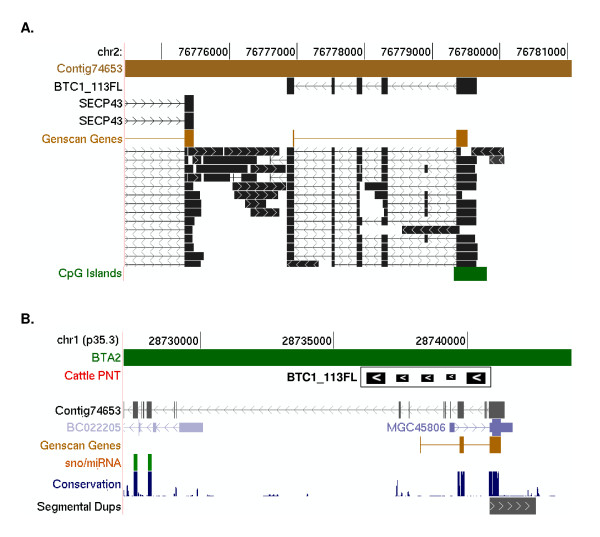
**Genomic context of BTC1_113FL**. Top panel (A). A modified image from the UCSC cow genome browser (March 2005, Btau_2.0) showing BTC1_113FL aligned to the cow genome (Contig74653; GenBank accession no. AAFC02073929). A track for cattle ESTs ("squish mode") shows high support for alternatively spliced cattle transcripts encoded in this region and the presence of 3' exons of *SECP43*. Genscan predicts a gene within the alignment of BTC1_113FL but does not predict all of the exons from ESTs. A CpG island is located at the 5' end of the predicted gene. Bottom panel (B). Anchoring of BTC1_113FL represented as a *block arrow*, to HSA1p35.3 (May 2004, Hg17). BTC1_113FL, was anchored on the basis of its complete alignment to cattle contig74653 and similarity of flanking sequence in the contig to the human genome (shown as vertical lines along the track). This region is syntenic to a segment of BTA2 (shown as a separate track), consistent with RH mapping data [23]. UCSC Genome Browser tracks for known human proteins, Genscan genes, sno/miRNA, conserved sequences, and segmental duplication are unmodified.

It was also possible to predict locations where genes encoding the NTs *should be *in the human genome (Additional file 2; Figures 2, 3, 4). This was accomplished by identifying the cattle genome sequence flanking the NTs with significant nucleotide similarity in the human genome (although the NTs themselves did not match the human genome using a BLASTN and TBLASTX E-value threshold of 10^-10^). Anchoring the NTs to the human genome using conserved flanking sequences revealed that 69% have an assumptive location in intergenic regions or within an intron of a known human gene. The genomic context for all 91 NTs is given in Additional file 2. A detailed description of the genomic context of three NTs is presented below. The number that can be presented is limited by available space (all alignments can be found in Additional file 5).

#### BTC1_14RD and BTC1_130FL: *alternatively spliced mRNAs of a novel, paralogous gene residing amongst known transcription factors*

BTC1_14RD (GenBank:XM_611254) and BTC1_130FL have 93% and 92% nucleotide identity, respectively, to *artiodactyla-specific transcript 1 *(*Ast1*) identified by Kim and coworkers [25]. Neither gene has an ortholog in the human or mouse genome. The three transcripts, Ast1, BTC1_14RD and BTC1_130FL, were aligned to BAC AC146804 (Figure 2; Additional file 6). Both BTC1_14RD and BTC1_130FL mapped at position BTA18:55591560–55600453, 5.4 kb from *Ast1 *(Figure 2). Genscan [26] also predicts a cattle gene in this region, which is supported by alignment of numerous cattle ESTs and a CpG island flanking the transcription start site (Figure 2). From the alignments it is apparent that BTC1_130FL is an alternatively spliced form of BTC1_14RD and that both transcripts represent a gene that is paralogous (>90% similar) to *Ast1 *(Additional file 6). The assumptive human genome context of BTC_14RD and BTC1_130FL was then investigated by anchoring to the human genome, conserved sequences flanking the NTs in the cattle BAC AC146804 (on BTA18). Nucleotide similarity was used to anchor BTC1_14RD, BTC1_130FL and *Ast1 *mRNAs to HSA19q, between the human genes *ZNF71 *and *ZIM2 *(Figure 2). This region of the human genome is rich in retrotransposed sequences, imprinted genes (*PEG3 *and *Zim2*) and genes encoding zinc-finger proteins thus indicating a high level of evolutionary and biological activity.

#### BTC1_146JE: a putative non coding RNA

BTC1_146JE is a 2206 bp transcript that contains an inverse repeat, and is found only in cattle and bottleneck dolphin (cetartiodactyl-specific). There is no ORF and no BLAST hit to any non-cetartiodactyl exon within 5 kb of flanking genomic sequence 5' and 3' of the NT gene on BTA21 (Figure 3; Table 3). A large number of unannotated, spliced cattle ESTs align to contig54150 in the same position further supporting its characterization as a novel spliced transcript.

The genomic DNA sequence flanking BTC1_146JE in contig54150 permitted comparative anchoring to HSA14q32.31 in an intergenic, non-conserved region containing a cluster of small nucleolar RNAs (snoRNAs; Figure 3). These snoRNAs are encoded in the introns of the non-coding maternally expressed gene *MEG8 *[27]. *MEG8 *is currently not included in the UCSC [28] database of known genes and thus not shown in Figure 3. To test the possibility that BTC1_146JE represents the pre-processed RNA for a snoRNA, a BLASTN search was carried out using the cluster of snoRNAs as query sequences (word size 7 and E-value threshold of 0.01). The 71 bp snoRNAs 14q(II-8) and 14q(11-9) [27] aligned with BTC1_146JE with short matches (23/26 and 27/30 identities, respectively). This suggests that BTC1_146JE represents a precursor RNA from which a cetartiodactyl-specific snoRNA (or miRNA) is processed.

#### BTC1_113FL: a novel transcript expressed preferentially in the thalamus

BTC1_113FL (GenBank:XM_611248) is a 659 bp transcript found only in cattle. It encodes a hypothetical protein 69 aa in length and whose gene is located on BTA2 (Table 3). Alignment of BTC1_113FL to cattle contig74653 reveals a gene with five exons (Figure 4). The gene is located within 1.5 Kbp of the 3' end of and in opposite orientation to *SECP43*. The presence of a gene in this location is strongly supported by a large number of spliced ESTs containing at least one GT/AG splice site, a Genscan prediction of 2 exons, a CpG island that spans the first exon and the probable transcription start site (Figure 4), and detection of expression in multiple tissues. DNA sequence flanking the gene corresponding to BTC1_113FL on contig74653 anchors it to HSA1p35.3, consistent with the available comparative mapping data [23]. Visual inspection indicates that BTC1_113FL should fall in a region of the human genome occupied by MGC45806 (GenBank:NM_152304), a RAB GTPase oncogene involved in vesicle-trafficking; however, BLASTN, TBLASTX and TBLASTN searches of BTC1_113FL against MGC45806 sequence resulted in no significant matches, demonstrating a lack of detectable homology between the exons of the two transcripts. BLAT [28] alignment of this NT against the human genome also reveals no alignment anywhere within the chromosome cytogenetic band where the NT is anchored by its flanking sequence. These data provide evidence that BTC1_113FL is a lineage-specific novel transcript.

### Expression and tissue distribution of NTs

**Figure 5:**
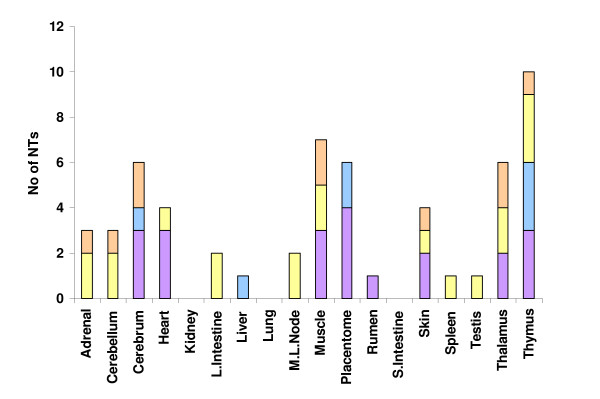
**Preferential expression of 39 NTs in 18 sampled tissues**. Orange bars indicate NTs with no ORF and preferential expression in more than one tissue. Yellow bars indicate NTs with ORFs and preferential expression in more than one tissue. Blue bars represent NTs with no ORF and preferential expression in a single tissue. Purple bars represent NTs with ORFs and preferential expression in a single tissue. M.L.; mesenteric lymph node.

Expression levels of the NTs were analyzed in 17 tissues from a one week-old Jersey calf and a term placentome. Expression of 86/91 NTs could be analyzed (5 had no representative cDNA element on the microarray). The expressed NTs were categorized with respect to the presence or absence of ORF(s) as well as with expression levels classified as high, moderate or low on the basis of an arbitrary scale (Table 5). Among the 60 expressed NTs with ORFs, 55% were expressed at a low level in all 18 tissues, and 45% were expressed at moderate or high levels in one or more tissues (Table 5). A similar distribution of expression levels in tissues was found among the NTs without ORFs. Tissue-preference in expression patterns of NTs was analyzed further by determining those NTs that were expressed greater than two-fold in any one tissue compared to at least 13 out of 17 other tissues (Figure 5; Additional file 2). A total of 39 NTs show tissue preference in their expression pattern. Of these, 28 were preferentially expressed in a single tissue. Six NTs were preferentially expressed in placentome, of which two were predicted to be ncRNAs. Ten different tissues showed exclusive expression of one or more of the NTs, with placentome and thymus having the largest number.

## Discussion

Comparative genomics, bioinformatics and microarray analysis were used to identify 91 transcripts encoded in the cattle genome, but not encoded in the genomes of non-ferungulate mammals (see further discussion below concerning the dog genome). The yield of NTs from the original EST collection is 91/12,614 = 0.8%, suggesting that NTs are relatively rare. Among the 91 NTs, 78 ORFs were identified, of which 48 are <100 codons and 30 are >100 codons (Figure 1; Additional file 3). The latter have a high probability of coding for a protein [29]. Using InterProScan [30], the lack of Pfam HMM matches for 99% of NT ORFs is strong evidence for absence of homology to known proteins. Anchoring the NTs to the cattle genome allowed identification of 30 transcripts having CpG islands upstream of their start sites (Additional file 2), providing additional support for their classification as protein-coding genes or ncRNAs. Specific protein functional motifs were identified in 10 predicted proteins encoded by the NTs, and 29 have functional non-coding motifs (Table 2; Additional file 2). All but five of the NTs showed evidence of active transcription in one or more tissues, and six were found to be preferentially expressed in cattle placentome, which is the source tissue of the EST collection used to mine for NTs. These results collectively provide the first conclusive evidence for an abundance of lineage- and tissue-specific transcripts encoded in the cattle genome.

The 21 NTs identified as high-probability ncRNAs provide a useful set of probes for exploring gene regulation in placental development and function [5,6]. Five of the putative ncRNAs are preferentially expressed in tissues that comprise the brain-immune-endocrine axis; placentome, thymus, thalamus, cerebrum or cerebellum. Seven contain inverted repeats that may be involved in internal base-pairing and gene regulation [31]. These putative ncRNAs may thus represent spliced, single-exon, primary snoRNA or microRNA transcripts [6]. Although microRNAs (miRNAs), snoRNAs and other ncRNAs that are not polyadenylated, may have been filtered out using our methods, on the basis of our findings, it is clear that placenta is a rich source of ncRNAs. Further studies are needed to clarify their functions in placental physiology.

The availability of a draft of the cattle genome sequence provided an opportunity to study the comparative genomic organization of the NTs, to confirm their authenticity, and to distill evidence for their origin, evolution and function. For the examples presented, as well as others in the dataset, the genes encoding the NTs are flanked by genes that are conserved in the human genome. This allowed us to identify a presumptive human genome context for the NTs. The recent availability of a 7.6× draft of the dog genome sequence allowed us to ask *ex post facto *whether the NTs are present in the dog genome. It was interesting to find that only five of the NTs (BTC_55FL, BTC1_102FL, BTC1_390NG, BTC1_21PW, and BTC1_40RD) matched sequences in the dog genome, suggesting that the sequences encoding these NTs were present in a ferungulate ancestor. The most parsimonious explanation for the presence of NTs in the cattle genome but not in other non-ferungulate mammalian genomes is that the NT genes were deleted from a common ancestor of primates and rodents after its divergence from the ferungulates (assuming that these five NTs will be found in other ferungulate genomes as well). Thus, in total, 65 NTs were identified that are (to date) only found in cattle, and 21 were identified in cattle and other cetartiodactyla (Table 4). Among the ruminants, this could represent the *de novo *formation of genes by overprinting [32], more recent gene deletion, extreme divergence, or the lack of complete genome sequence information for the other species. Other proposed mechanisms for the appearance of lineage-specific genes, such as retrotransposition [reviewed in [33]], are not excluded by our analysis. Further study of the phylogenetic distribution of the NT genes will provide a better understanding of their origin, and the timing of gene loss/gain in ancestral species.

The cetartiodactyl NTs BTC1_14RD and BTC1_130FL represent interesting examples of transcripts that are encoded within a highly dynamic genomic context (Figure 2). *BTC1_14RD*/*BTC1_130FL *and *Ast1 *(artiodactyl-specific transcript1) are located in a region of the cattle genome that is surrounded by genes encoding zinc-finger proteins. *Ast1 *and its neighboring genes, *PEG3 *and *ZIM2*, were previously shown to undergo lineage-specific imprinting, and *PEG3 *and *ZIM2 *are thought to have undergone rearrangements independently in different lineages [25]. Roughly 80 Kbp of the cattle genome between *PEG3 *and *ZIM2 *(BTA18:55,580 Kbp – 55,655 Kbp) spanning BTC1_14RD and *Ast1 *is absent in the human genome, and there are a large number of segmental duplications in this region. The entire locus maps ~1 Mbp from the telomere of BTA18. It is known that telomeric and subtelomeric regions are highly active in segmental duplications and the formation of novel genes [34]. Thus, the artiodactyl-specific genes for Ast1 and BTC1_14RD appear to have been created by a segmental duplication. Analysis of other ferungulate genomes will shed greater light on the origin and evolution of these interesting genes. Given their genomic context and imprinting status these genes may play an adaptive role in placental function.

The significant number of novel transcripts expressed preferentially in cattle placenta and in other tissues raises the question of their role in adaptive evolution. Are the genes encoding these transcripts lost in other genomes because they are dispensable or do they have adaptive evolutionary significance? Is gene gain by insertion, segmental duplication and/or chromosome duplication a driving mechanism of adaptive evolution? Evolutionary theory has long held that adaptive phenotypic change is realized through changes in developmental processes [35]. The genes that control these developmental processes are known to be highly conserved [36] and it is believed that adaptive evolutionary change is fueled by mutations that modify the expression of these conserved regulatory loci [36,37]. We and others have proposed that rapidly evolving genes, "novel" or lineage-specific genes, and ncRNAs play a role in mediating changes in gene expression that affect regulatory genes controlling fundamental developmental processes [13,38-40]. Lineage-specific genes can appear as a result of non-homologous recombination of exons between different genes [32,41-43], or from fast-evolving duplicated genes that have lost significant sequence similarity even within relatively short evolutionary time-spans [44,45]. Keese and Gibbs [32] provided numerous examples of genes that are created *de novo *by translation of previously unused reading frames of existing coding and non-coding genomic DNA, a mechanism that has been called "overprinting" [46,47]. Each of the NTs has its own evolutionary history, and understanding their origins will require sequence information from additional mammalian genomes. Whether they are rapidly evolving and dispensable [48] and/or have adaptive functions can only be determined by experimentation [37].

## Conclusion

The 91 lineage-specific transcripts discovered in the present study are a new resource for studies of adaptive changes in placental architecture and function. The tissue distribution of the NTs suggests that many of them also have adaptive roles in other tissues. The presence of so many lineage-specific genes in cattle and their association with subtelomeric regions, which are hotspots for chromosome rearrangements and recombination, suggests that gene loss and gain are important mechanisms of genome evolution in mammals. The sequencing of additional vertebrate genomes with defined phylogenetic relationships will enable the search for lineage-specific genes to take on a more evolutionary perspective that is required to understand their origins and functions.

## Methods

### Identification and characterization of novel transcripts expressed in cattle placenta

A collection of 12,614 5' ESTs from a normalized and subtracted cattle placenta cDNA library was selected as an EST resource for novel transcript discovery [49]. The repeat-masked ESTs were analyzed for similarity to non-cetartiodactyl ESTs and genomic sequences using BLASTN [15] at a threshold E (expectation value) <10^-05^, and NCBI dbEST and non-redundant DNA sequence databases from which cetartiodactyl sequences were removed. All ESTs that matched non-cetartiodactyl sequences at E < 10^-05 ^were removed from the starting set (Table 1). Another BLASTN search against human draft sequences at E < 10^-10 ^removed additional ESTs from the set. The entire process of searching, parsing, and subtraction was carried out iteratively with a set of pipelined Perl scripts (PipeBLASTN).

PipeBLASTN was followed by *in silico *extension of the remaining placenta ESTs using public domain cattle ESTs. The ESTs were extended using a custom clustering algorithm and the CAP3 [50] assembly program. The clusters were created using stringent parameters (minimum overlap length of 40 bp and 95% minimum sequence identity). CAP3 was run with default parameters, on each EST cluster. The *in silico *extended ESTs were run through PipeBLASTN again to remove any non-cetartiodactyl homologs. The in-house source clones of the remaining 5' extended ESTs were sequenced in the 3' direction using an anchored oligodT primer. The 3' ESTs were vector- and quality-trimmed, repeat-masked, and assembled to their 5' extended counterparts to obtain full-length clone sequences. Those 5'-3' pairs that did not overlap were subjected to additional rounds of primer-walking until complete sequences were obtained.

To extend the newly assembled sequences further the process of clustering and assembly with public domain cattle ESTs followed by PipeBLASTN was carried out again to yield a set of putative NTs and divergent orthologs. The assembled sequences were manually edited and proofed for spurious assemblies, un-called bases, and reverse-complementarity. The divergent homologs were separated from the putative NTs by interrogating the sequences for distant homology in other species using BLASTX against non-redundant proteins (NCBI, April 2006), and TBLASTX against human and mouse UniGene (Build 190 and 152, respectively) and ESTs from other species (NCBI, April 2006). Sequences that aligned to database sequences below an empirically chosen E-value threshold of 10^-10 ^were designated as putative divergent homologs [12], and those that scored above this threshold were designated NTs. The NTs with similarity to cetartiodactyl-specific genes were identified by BLAST against a database of cetartiodactyl-specific non-redundant sequences and ESTs. Sequence data from this article have been deposited with the DDBJ/EMBL/GenBank data libraries and the accession numbers are listed in Additional file 2.

The second assembly (March 2005, Btau_2.0; BCM-HGSC) of the 6.2× cow genome draft sequence [17] was used to determine how many NT source clones were primed from an internal polyA tract during their sequencing from the 3' direction with an anchored oligodT primer. To do this, each NT contig was manually checked to ensure the terminal positioning of the 3' EST within the contig. The NTs were then aligned to the scaffold sequence. The aligned region of the scaffold and flanking 100 bp was extracted for each of the aligned NTs. If the 3' EST of a NT was primed by an internal polyA tract, it would be visible in the scaffold downstream from the 3' end of the aligned NT. These NTs were removed from the set. In addition, trimming of low-quality sequences from 3' ESTs may have resulted in complete or partial removal of the polyadenylation signal. Each of the corresponding scaffold sequences was scanned for the presence of a polyadenylation signal within 50 bp downstream from the end of an aligned NT.

#### Identification and characterization of ORFs

High-probability ORFs were identified in the NTs using NCBI's ORFfinder [51]. The locations of start and stop signals were determined in all the three reading frames for NTs with a known polyadenylation signal, and in all six reading frames for NTs with no identifiable polyadenylation signal. The most probable complete or longest partial ORFs were selected using a threshold length of 100 bp. The manual analysis of all the six reading frames involved choosing the longest ORF that either contained more than one downstream stop codon, or one or more juxtaposed downstream start and stop signals within 20 codons of each other. The selected ORF contained at most two upstream stop signals, and one upstream ORF < 30 codons in length. The use of a six-frame analysis to select ORFs that were clearly demarcated by strong start and stop signals helped to reduce the probability of picking false ORFs even though we used 33 codons as a threshold. The complete ORFs that had a start and a stop codon were analyzed for the presence of the Kozak consensus, the eukaryotic translation start site context [19]. False positives in ORFs were predicted by randomizing the NT and then predicting ORFs in these sequences using the same rules for ORF prediction [52]. Because ORFS between 33 and 100 codons are predicted to have ~50% false positive rate [52], all ORFs >33 codons were considered as candidates for encoding small novel proteins. The sequences of the NTs and their predicted proteins can be found in Additional files 1 and 3.

The ORFs were analyzed for protein motifs and domains using *InterProScan *[30]. Only those motifs with a precision rate >93% were reported [53]. Additionally, ORFs were searched for transmembrane helices using *TMHMMv2.0 *[54] and *SVMtm *[55], and for signal peptides using *SignalP *with both neural networks and hidden Markov models [56]. Conflicting predictions of a signal peptide and a transmembrane region in the same sequence were resolved as follows: i) if the prediction was within 15 codons from the 5' end then the prediction was counted as a signal peptide; ii) if the signal peptide was predicted anywhere else, *Phobius *[57] was used to confirm either prediction.

The ORFs were searched for novel repeated protein domains using the methodology and software described by Yeats et al. [58]. *Prospero *was used to self-search the ORFs for occurrence of internal duplications. Alignments with a length of less than 30 residues (threshold length of a protein domain) were removed. To search for repeated domains, alignments in which the start points of each sub-sequence were separated by less than 45 residues were discarded. The alignments generated by *Prospero *were used as an initial alignment to make profile Hidden Markov models using HMMER [59], and the resulting alignments were scanned against the Swiss-Prot database.

#### Characterization of non-coding regions

The NT sequences were scanned for functional UTR elements using *UTRScan *[60]. To identify UTR elements that may occur in NTs by chance, the NTs were randomized using the program *shuffle *in SQUID software [61], and searched for motifs using *UTRScan*. This was repeated 10 times. The observed frequency of a motif was compared to that found in the randomized NT sequences. The motif was reported if its observed frequency was at least 4 times greater in the NTs as in the randomized NT sequences. The GC-content of each sequence was estimated with the program *geecee *from the EMBOSS suite [62]. Candidate CpG islands in 5' exons and non-coding regions were identified by aligning NTs to the cattle genome. The aligned scaffold region and 200 bp upstream from the start of the alignment were extracted. These sequences were searched with the command-line version of *CpG Island Searcher *[21] using a minimum CpG island length of 200 bp, minimum GC-content of 53%, and observed/expected CpG ratio of 0.63. The choice of parameter values was based on the averages found by Takai and Jones [21] for exonic CpG islands. Additionally, each NT sequence was searched against itself to identify IRs and confirmed independently using the *einverted *program [62].

The NTs were analyzed for guanine-rich sequence motifs capable of forming three-dimensional structures called G-quadruplexes or G-quartets [22]. On the basis of the algorithm by D'Antonio and Bagga [63], a program was written to identify the G-quartet motif in the NTs. The results were compared with results obtained using shuffled sequences as a control. *QRNA *[64] was used to search for conserved ncRNA structures amongst the NTs. The input to *QRNA *was a file containing the search results from a BLASTN of NTs against the non-redundant DNA sequence database. Default parameters were used, and the results were compared with another run of *QRNA *using shuffled data as a control.

### Microarray expression analysis

The gene expression data for the NTs was extracted from a larger microarray data set obtained by profiling total RNA from 17 different tissues collected by vivisection of a one week-old Jersey calf (NCBI Gene Expression Omnibus series entry GSE3029) and term placentome. Expression patterns were obtained using a microarray containing approximately 7000 cattle placenta cDNAs spotted in duplicate on glass slides [65]. The expression profiles for each tissue were obtained by comparing expression of each gene to a reference standard comprised of RNAs from bovine brain and three different cell lines [65]. In order for a gene to be included in the analysis, its fluorescence intensity had to be greater than three standard deviations (SD) above the background in either the sample or reference standard. All but five of the NTs were represented on this array by a cDNA clone.

The NTs were further categorized based on their fluorescence intensity values. Expression was considered as "high" if the fluorescence intensity was greater than 5000 +3SD above background in at least one tissue, "moderate" if fluorescence intensity was between 1000 and 5000 in at least one tissue, and "low" if the fluorescence intensity was between 50 and 1000 +3SD above background in all 18 tissues.

To determine the number of transcripts that were preferentially expressed in the eighteen tissues, the ratio of the normalized intensity ratio was calculated for every pair of tissues (ratio-of-ratios) using a Perl script. For a transcript to be considered as preferentially expressed in a given tissue *t*, the following relationship had to hold true in at least 13 out of 17 other tissues:

R (*t*)/R (any other tissue) > 2,

where R is the normalized intensity ratio (tissue:reference).

### Analysis of the cattle and assumptive human genome context of NTs

The number of NTs with matches in the cattle genome and those showing evidence of splicing were determined using a BLASTN search against all cattle genome sequences, including the draft assembly (March 2005, Btau_2.0, BCM-HGSC; and NCBI BACs). The NTs having evidence of "split matches" were analyzed using *est2genome *[62] in order to determine exon-intron boundaries. An NT was classified as spliced if, after alignment to genomic sequence, the intron-exon boundary had a GT/AG splice site.

To identify candidate ncRNAs amongst the NTs that lacked an ORF, the NTs were aligned to the cattle genome assembly. Whenever possible, the aligned region and 5 kb of flanking sequences was extracted from each of the aligned scaffolds. The extracted region was searched for the presence of exons by doing a BLASTX search against Swiss-Prot and non-redundant protein databases using an E-value threshold of 10^-03^. All NTs that lacked an ORF and aligned to sequence scaffolds with no BLASTX hits were characterized as candidate ncRNAs.

The NTs were mapped *in silico *to the cattle genome using BLASTN search of the NTs against the cattle genome assembly and repeat-masked cattle genome scaffolds (March 2005, Btau_2.0, BCM-HGSC). The scaffolds containing the NTs were searched for similarity to the human genome, (NCBI Build 35; E < 10^-05^). The chromosome number, start and end positions, and orientation were parsed for the top human BLASTN hit to the cattle assembly sequences (Additional file 5). Custom tracks were generated using these data, and loaded onto the UCSC Genome Browser [66,28]. Using the cattle-human comparative map [23] also available on the UCSC Human Genome Browser, the position of the NTs on cattle chromosomes could be accurately placed. The cattle-human comparative map was also used to identify NTs that were located within 1 Mbp of evolutionary breakpoints, and within 2 Mbp of telomeres. The distribution of the 91 NTs was compared to the distribution of 91 random *RefSeq *genes by simulation as performed by Murphy et al. [67]. Chi-square test was performed to determine if NTs are associated with subtelomeric regions.

In order to estimate where the genes encoding the NTs would be if they were present in the human genome, conserved sequences in cattle contigs flanking each NT were used to anchor them in the human genome. The orientation and distance of the NT in the cattle assembly was used to juxtapose the NT in the human genome sequence. The annotation of each NT in the cattle and human genomes can be found in Additional file 2.

## Note added in proof

The authors recognize that the annotation of the 91 NTs may change with updated versions of RefSeq and other genome databases. A table containing updates to the annotation of these sequences will be available at the Laboratory of Mammalian Genome Biology website [68].

## Authors' contributions

CGK participated in the design of the study, wrote the computer programs for the pipeline, EST clustering, expression and genome analysis, conducted the sequence analysis and interpretation of data, and drafted the manuscript. JHL participated in the design of the study, and assisted in the collection and analysis of DNA sequence data. MRB contributed microarray data. HAL supervised the research, participated in the design of the study, interpretation of data and drafting of the manuscript. All authors have read the manuscript, provided critical reviews of content, and approved the final manuscript.

## Supplementary Material

Additional file 1 FASTA formatted full-length clone sequence for 91 NTs Click here for file

Additional file 2 Feature table for 91 NTs Click here for file

Additional file 3 FASTA formatted predicted protein sequence for 64 NTs Click here for file

Additional file 4 **Secondary structure of the class 2 SECIS element identified in the NT BTC1_43PW**. The element contains a characteristic conserved SECIS core followed by an 11 bp stem. All the conserved bases are shown in bold. Click here for file

Additional file 5 Cattle genome BLAT alignments to NTs, and cattle contig BLAT alignments to human genome Click here for file

Additional file 6 **Alignment of BTC1_14RD, BTC1_130FL and Ast1 transcripts to cattle genomic sequence**. A) Pipmaker [69] output depicts the alignment of the three transcripts to the cattle BAC sequence AC146804 [25]. The artiodactyl-specific transcript, *Ast1 *[GenBank:AY427788] [25], is organized as 4 exons. The NT BTC1_14RD is organized as 7 exons. *Ast1 *has a predicted ORF length of 152 codons and BTC1_14RD has a predicted ORF length of 235 codons. B) Pipmaker output depicts the alignment of BTC1_130FL, an alternate spliced variant to BTC1_14D, to cattle BAC sequence AC146804. The transcript is organized as 3 exons, and has an ORF length of 135 codons. Click here for file

## Figures and Tables

**Table 1:** Bioinformatics scheme for identifying novel transcripts

Steps in the scheme	Number of sequences	
	Removed	Remaining
Starting set of 5' placenta ESTs		12,614
PipeBLASTN (automated BLASTN analysis)	10,235	2,379
*In silico *extension of 5' ESTs with public cattle ESTs, followed by PipeBLASTN	581	1,798
3' reads from single-pass sequencing using anchored oligodT primer	618	1,180
PipeBLASTN of 3' reads	404	776
Assembly of 3' and extended 5' mates	NA^a^	493
Full-clone sequencing of unassembled 3' ESTs	NA	283
*In silico *extension of 493 mate-pairs followed by PipeBLASTN	171	322
*In silico *extension of 283 primer-walked clones, followed by PipeBLASTN	232	51
Working set of divergent homologs and novel transcripts (322+51)	NA	373
TBLASTX against UniGene databases to separate novel transcripts from divergent homologs	75	298
*In silico *extension followed by PipeBLASTN using updated databases to yield final set of NTs.	134	164
Screening for internal poly A tracts	73	91

^a ^NA; not applicable.

**Table 2:** Evidence of functionality in 91 NTs^a^

Type of functional element	No. of NTs
Protein motif	4
Coiled-coil repeats	1
Transmembrane (TMHMM, SMART)	1
Novel protein domain	1
Signal peptide (SignalP-NN, SignalP-HMM, SMART)	4
UTR elements	13
Inverse repeats	12
G-quartets	7
CpG Islands	30
Non-coding RNA structural elements	2
ncRNA	21

^a ^Sequences annotated with the above elements can be found in Additional file 2.

**Table 3:** Sample feature table for 91 NTs

**NT Id^a^**	**GenBank accession^b^**	**length (bp)**	**ORF_length aa^c^**	**species^d^**	**No. exons**	**poly A signal^e^**	**motif^f^**	**tissue preference**	**Hg17 locus**	**BTAU_2.0 assignment**	**Cattle genome context^g^**	**Segmental location^h^**
BTC1_14FL (XM_873204)	CK394134	868	63		4	AATAAA	CpG	muscle	7p13	chr4:43, 188,679–43,191, 770	intergenic	interstitial
BTC1_15FL	AY563860	630	none	C	1	AATAAA	ncRNA	none	14q11.2	scaffold3389:59,086–59,711	inter genie	interstitial
BTC1_33FL	CK394175	691	35	C	5	ATTAAA	CpG	none	6q14.3	scaffold4626:20,602–22,398	intergenic	interstitial
BTC1_34FL	CK394179	977	100 K	A	3	AATAAA	ADH_DRE, GY-Box, signalP	placentome	9q32	scaffold9964:10,570–17,798	intergenic	interstitial
BTC1_53FL	AY563751	525	106	C	1	AATAAA	none	none	19q13.31	chr6:49, 142,506–49,143,026	intergenic	interstitial
BTC1_55FL	AY563756	777	82 K	A	2	AATAGA	signalP	none	16q22.1	chr18:13,861,852–13,862,619	intergenic	boundary
BTC1_57FL	AY563869	911	none	C	1	AATAAA	none	thymus	7p22.1	chr25:33,360,786–33,361,318	*ZnF447*	interstitial
BTC1_58FL	AY563762	1544	64	A	2	ATTAAA	ADH_DRE, GY-Box	none	11q12.2	chr29:31,601,481–31,607,081	*RAB3*	interstitial
BTC1_71FL	AY563803	691	none	C	1	AATAAA		none	3p21.31	chr22:42,680,522–42,681,188	*SACMIL*	interstitial
BTC1_77FL	AY563824	1095	84	C	1	AATAAA	GY-Box; CpG	thymus	18q21.33	chr24:44,739,526–44,740,613	*HDGFRP2*	subtelomeric
BTC1_78FL	CK394028	1736	91 K	A	1	AATAAA	GY-Box; IR	mesenteric lymphnode, thymus	1p36.13	chr2:85,996,428–85,997,304	intergenic	subtelomeric
BTC1_79FL	AY563826	732	none	C	4	TATAAA	CpG; ncRNA	cerebrum	2p11.2	chr11:34,570,339–34,575,174	intergenic	interstitial
BTC1_92FL (XM_587188)	CK394038	933	93 K; 127	C	1	TGTAAA	none	cerebrum	17q25.1	chr19:49,709,796–49,710,719	intergenic	interstitial
BTC1_93FL	AY563890	1269	147 K	C	1	AATAAA	GY-Box	thymus	22q12.2	chr17:42,667,501–42,668,530	*SEC14L4*	interstitial
BTC1_95FL	AY563713	2492	108	C	1	AATAAA	none	skin	6p21.2	chr23:8,629,884–8,632,373	intergenic	interstitial
BTC1_102FL	AY563714	795	38 K	C	1	AATAAA	G-quartet; IR	none	10q11.21	chr28:33,147,612–33,148,436	intergenic	subtelomeric
BTC1_113FL (XM_611248)	CK394057	659	69 K	C	5	AATAAA	CpG	thalamus	1p35.3	chr2:76,776,847–76,779,658	intergenic	interstitial
BTC1_115FL	CK394060	962	36; 35	C	1	AATAAA	none	thymus	1p34.1	chr3:67,576,355–67,577,638	*LRRC41*	interstitial
BTC1_130FL (XM_611254)	CK394181	664	125 K; 65 K	A	3	AATAAA	none	adrenal, cerebellum, thalamus	19q13.43	chr18:55591568–55593819	*ZIM2*	subtelomeric
BTC1_132FL	CK394199	2174	none	A	1	AATAAA	IR; ncRNA	thymus	Xq25	scaffold4717:48,263–50,399	*ERVK6*	interstitial
BTC3_7JE	CK393999	548	35	A	5	AATAAA	none	muscle	7p22.1	chr4:43,188,676–43,191,750	intergenic	interstitial
BTC1_22JE	CK394011	1569	none	A	1	ATTAAA	IR; ncRNA	placentome	5q33.3	scaffold399:92,975–94,544	*TIMD4*	interstitial
BTC2_43JE	CK394035	1043	none	C	2	AAACAA	G-quartet; CpG	cerebrum, thalamus	8q24.3	scaffold12671:9,483–10,533	*JRK*	interstitial
BTC1_51JE	CK394043	958	130	C	2	AATAAA	none	skin	11p15.5	scaffold2079:18,521–19,645	intergenic	interstitial
BTC1_100JE	CK394097	2381	92 K	C	1	GATAAA	GY-Box	none	19p13.11	chr7:5,772,691–5,775,068	intergenic	interstitial
BTC1_104JE (XM_883284)	CK394101	619	59; 68	A	5	AATAAA	None	placentome	none	scaffold6598:22994–33093	intergenic	interstitial
BTC1_113JE	CK394107	830	none	A	2	AATAAA	CpG; ncRNA	none	1p13.2	scaffold1237:214289–215207	intergenici	interstitial
BTC1_118JE	CK394113	2257	50; 92	C	1	AATAAA	GY-Box	none	22q13.2	scaffold9684:8273–9215	intergenic	interstitial
BTC1_144JE	CK394138	832	150 K	C	1	AATAAA	none	none	1p36.33	chr16:37642343–37643174	intergenic	boundary
BTC1_146JE	CK394139	2206	none	A	3	ATTAAA	IR; ncRNA	muscle, skin	14q32.31	chr21:45,674,164–45,678,931	intergenic	interstitial
BTC1_203JE	CK394182	773	129 K	C	3	ATTAAA	CpG	heart	8q21.2	chr14:46,885,318–46,890,350	intergenic	interstitial
BTC1_215JE	CK394201	1038	109 K	C	1	AATAAA	Eu_thiol protease	none	2p25.3	scaffold473:40,328–41,349	intergenic	interstitial
BTC1_8NG	AY563705	703	71 K	A	4	AATATA	none	thalamus	none	chr19:48,755,357–48,757,924	*ATP1a2a*	interstitial
BTC1_39NG	AY563711	767	172 K	C	2	AATAAA	Proline-rich with_coiled-coil	none	1p31.3	scaffold997:257,434–259,042	*INADL*	interstitial
BTC1_58NG	AY563715	562	49	C	1	AATAAA	CpG	cerebrum	13q34	chr12:48,063,584–48,064,091	*CDC16:*	subtelomeric
BTC1_63NG	AY563716	483	142 K	C	2	unknown	signalP; ATPaseE1. E2	none	16p13.12	chr25:14,893,445–14,897,173	intergenic	interstitial
BTC1_104NG	AY563722	728	144 K; 149 K	C	1	AATAAA	TOP	none	14q32.32	chr21:48,386,644–48,387,349	*MKNK2 *i	subtelomeric
BTC1_149NG	AY563726	690	35	C	1	TATAAA	CpG	skin, testis	22q11.21	chr17:45,366,139–45,366,804	*RFNG*	subtelomeric
BTC1_237NG	AY563754	622	67	C	1	AATAAA	CpG	cerebrum	17q25.3	chr19:47,237,560–47,238,117	intergenic	interstitial
BTC1_255NG (XM_596632)	AY563763	651	64	C	2	AATAAA	CpG;Heavy metal_ion transport	muscle	3q29	chr1:43,207,885–43,208,506	intergenic	interstitial
BTC1_269NG	AY563771	716	116	C	1	ATTAAA	CpG	none	11q13.3	chr29:41,407,248–41,407,965	intergenic	boundary
BTC1_286NG (XM_870778)	AY563780	788	36; 184 K	C	1	unknown	CpG	placentome	none	chr14:662,313–663,087	*MAPK8IP2*	subtelomeric

^a ^number in parenthesis is the GenBank accession of the cattle RefSeq prediction matching the NT.

^b ^GenBank accession No. of 3' sequence including primer-walked sequence.

^c ^K denotes start-codon of ORF flanked by Kozak consensus.

^d^C, cattle-specific; A, cetartiodactyla-specific

^e ^'Unknown' indicates that a polyadenylation signal could not be identified.

^f ^'IR' indicates presence of an inverse repeat; ADH_DRE, GY-box, K-box, Brd box and CPE are functional UTR elements.

gene symbol indicates NT is located in an intron of that gene.

^h ^"Boundary" indicates that the NT anchors within 1 Mbp of the end of a homologous synteny boundary on a human chromosome as defined in [23];

"Subtelomeric" indicates that the NT aligns within 2 Mbp from the end of a cattle chromosome.

**Table 4:** Lineage specificity and expression features of 91 cattle NTs

**Lineage specificity**	**Predicted ORF (>100 bp)**	**No. of NTs**	**Expression^a^**	**Evidence for transcript splicing^b^**
Cetartiodactyla-specific transcripts^c^	No	7	7	3
	Yes	16	14	15
Novel transcripts^d^	No	20	19	6
	Yes	48	46	18

^a ^Gene expression validated by microarray analysis.

^b ^Splice-site analysis was conducted using *est2genome*. Some of the NTs had missing exons due to gaps in the sequence scaffolds, or due to alignment reaching the end of a scaffold.

^c ^BLASTN hits with E ≤ 10^-05 ^to cetartiodactyl EST database.

^d ^Sequences that have BLASTN hits with E > 10^-05 ^and TBLASTX hits with E > 10^-10^

**Table 5:** Expression levels of 86 NTs

**Expression Level^a^**	**NTs with ORFs**	**NTs with no identifiable ORF**
High	12 (14%)	5 (6%)
Moderate	14 (16%)	10 (11%)
Low	33 (38%)	12 (14%)

^a ^See Materials and Methods for definition
