# Supplementary figures and images for: Discovery and characterization of 91 novel transcripts expressed in cattle placenta
Source: BMC Genomics. 2007 May 9;8:113. doi: 10.1186/1471-2164-8-113 (PMC1884150; doi:10.1186/1471-2164-8-113)

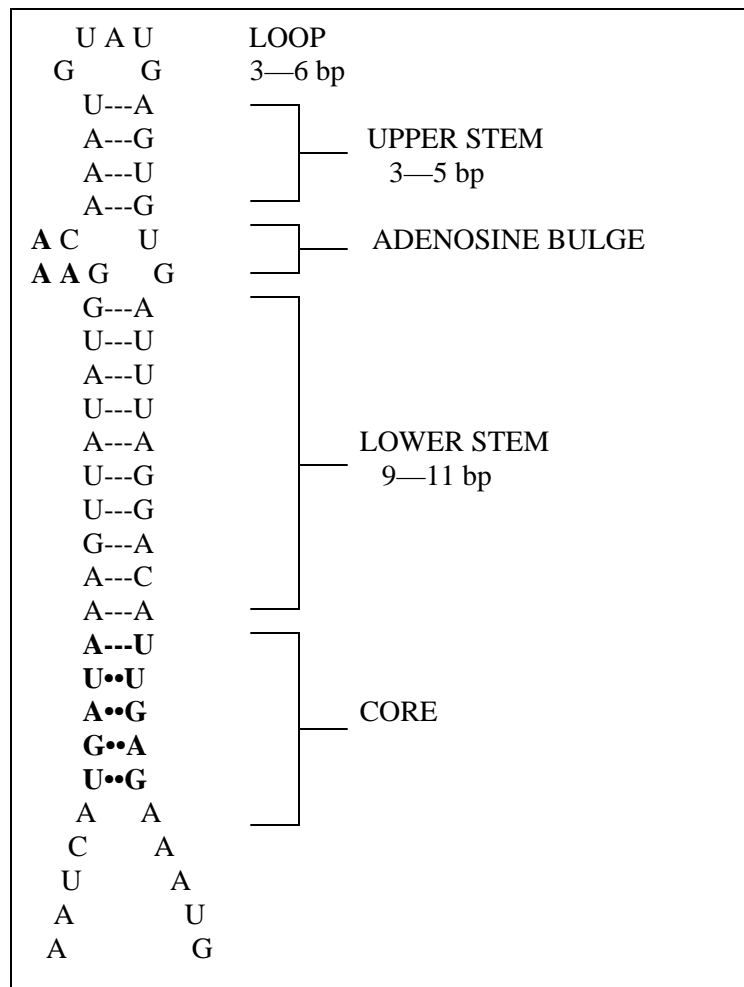

Supplement: Additional file 4 — Secondary structure of the class 2 SECIS element identified in the NT BTC1_43PW. The element contains a characteristic conserved SECIS core followed by an 11 bp stem. All the conserved bases are shown in bold. [file 1471-2164-8-113-S4.pdf]

**A.**


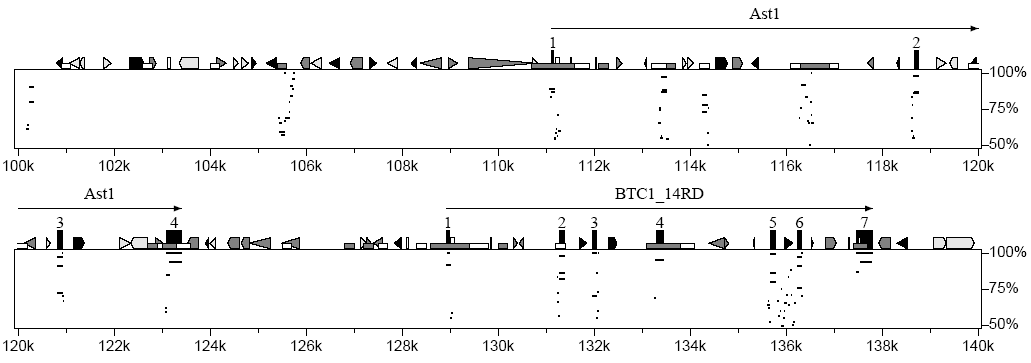


**B.**


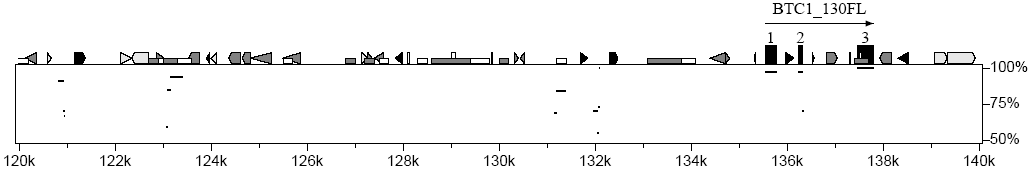

Supplement: Additional file 6 — Alignment of BTC1_14RD, BTC1_130FL and Ast1 transcripts to cattle genomic sequence. A) Pipmaker [69] output depicts the alignment of the three transcripts to the cattle BAC sequence AC146804 [25]. The artiodactyl-specific transcript, Ast1 [GenBank:AY427788] [25], is organized as 4 exons. The NT BTC1_14RD is organized as 7 exons. Ast1 has a predicted ORF length of 152 codons and BTC1_14RD has a predicted ORF length of 235 codons. B) Pipmaker output depicts the alignment of BTC1_130FL, an alternate spliced variant to BTC1_14D, to cattle BAC sequence AC146804. The transcript is organized as 3 exons, and has an ORF length of 135 codons. [file 1471-2164-8-113-S6.doc]
